# FAM46C as a Potential Marker for Pan-Cancer Prognosis and Predicting Immunotherapeutic Efficacy

**DOI:** 10.3389/fgene.2022.810252

**Published:** 2022-02-09

**Authors:** Jiehua Deng, Wei Xiao, Zheng Wang

**Affiliations:** ^1^ Centre of Imaging Diagnosis, Affiliated Tumor Hospital of Guangxi Medical University, Nanning, China; ^2^ Department of Clinical Medicine, Medical College of Shihezi University, Shihezi, China

**Keywords:** human cancer, immunomodulation, immunotherapy, prognosis, tumour microenvironment

## Abstract

**Background:** *FAM46C* is a common mutated gene in tumours. A comprehensive understanding of the relationship between *FAM46C* expression and pan-cancer can guide clinical prognosis and broaden the immunotherapeutic targets.

**Methods:** Data from The Cancer Genome Atlas and Genotype-Tissue Expression (GTEx) databases were obtained, and gene expression of different tumour types and stages was analysed. Immunohistochemical analysis was performed to detect differences in the *FAM46C* protein levels in normal and cancerous tissues. The genetic variation of *FAM46C* was characterised using cBioPortal. The clinical prognostic value of *FAM46C* and the impact of *FAM46C* expression levels on the prognosis of patients with different types of cancer were assessed based on Kaplan–Meier and Cox regression analyses. Gene set enrichment analysis (GSEA) was used to analyse the pathways associated with *FAM46C*. Correlations between *FAM46C* expression levels and immune infiltration were assessed using the TIMER2 database and CIBERSORT algorithm, and correlations between *FAM46C* expression and the ESTIMATE, immune and stromal scores were analysed using the ESTIMATE algorithm. In addition, we also analysed the correlation between *FAM46C* expression and immune activation, suppression genes and immune chemokines.

**Results:** The expression level of *FAM46C* was correlated with the prognosis of most tumours, and low expression levels often suggested a poor prognosis. *FAM46C* was positively correlated with the abundance of CD4^+^ T cells, CD8^+^ T cells and plasma B lymphocytes in the tumour microenvironment. *FAM46C* exhibited a strong correlation with immunomodulatory pathways, immunomodulatory factors and immune markers. In addition, high *FAM46C* expression correlated with tumour mutational burden in acute myeloid leukaemia and microsatellite instability in endometrial cancer.

**Conclusion:** Our study suggests that *FAM46C* can be a potential prognostic marker for pan-cancer, is closely associated with immune regulation and may be an immune checkpoint to guide future clinical immunotherapy.

## Introduction

Cancer is one of the major diseases threatening human health, and the incidence and mortality rate of cancer are increasing every year (Sung et al., 2021). The World Health Organization estimates that the number of cancer cases worldwide may increase by 60% in the next 20 years. Although significant progress has been made in cancer diagnosis and treatment in recent years, the overall survival (OS) rate of cancer patients has not improved significantly owing to limited research on the mechanisms of cancer occurrence and development (Siegel et al., 2021).

Some studies have demonstrated that mutations and deletions of cancer-related genes are the main cause of cancer development and progression (Clinton et al., 2020). Therefore, more in-depth studies are required, which may provide relevant clues and ideas for new therapeutic approaches. The Cancer Genome Atlas (TCGA) has characterised genomic and epigenomic variations in multiple classes of tumour cells. Based on such large data samples, multiple epigenomes can be sequenced and integrated for pan-cancer analysis, and the results can be correlated with clinical and imaging manifestations for comparison. Therefore, the origin and occurrence of tumours and the underlying mechanisms of tumour cell signalling pathways can be analysed. Previous studies have suggested that tumour cells originating from different organs may exhibit the same or similar histomorphological characteristics. For example, squamous cell carcinoma can originate from the skin, head and neck, oesophagus, cervix and vagina. Moreover, tumour cells originating from the same organ can also exhibit different histomorphological characteristics; the examples include thyroid and kidney cancers. Pan-cancer analysis facilitates the study of commonality and heterogeneity of tumours based on cellular and molecular characteristics and helps to assess molecular relationships among multiple tumours and understand their underlying regulatory mechanisms. It also provides new clues to further develop the clinical treatment of cancer.

The *FAM46* protein is a non-canonical poly(A) polymerase and a member of the nucleotidyltransferase (NTase) fold superfamily. It relies on the poly(A) polymerase activity to induce polyadenylation of the mRNA of target proteins, which may regulate antibody production, gene expression and development of malignant tumours in the body and induce apoptosis (Kuchta et al., 2016). It is mainly found in vertebrates, and four proteins (FAM46A/B/C/D) are mainly expressed in humans. Among these proteins, *FAM46C* was first identified as one of the most common mutated genes in patients with multiple myeloma (MM) and is associated with decreased OS in patients with MM (Zhu et al., 2017). Furthermore, recent studies have found a functional association between *FAM46C* and hepatocellular carcinoma (Zhang et al., 2017), gastric cancer (Tanaka et al., 2017), colorectal cancer (Zhang et al., 2020), oral squamous cell carcinoma (Zhuang and Lu, 2018) and prostate cancer (Ma et al., 2020). *FAM46C* may also inhibit tumour proliferation and metastasis and promote tumour cell apoptosis through several pathways (Kazazian et al., 2020). The absence of *FAM46C* is reported to be associated with recurrence and decreased OS after radical surgery for gastric cancer (Tanaka et al., 2017). In patients with pancreatic ductal adenocarcinoma, *FAM46C* may also mediate the development of autoimmune diseases by participating in the epidermal growth factor receptor (EFGR) and interferon (IFN) signalling pathways (Crow, 2010). However, the prognostic accuracy of *FAM46C* in various tumours and its role in immune regulation remain unclear.

The present study provides a comprehensive analysis of *FAM46C* expression in pan-cancer based on the data from TCGA, Oncomine and other databases. We analysed differential gene expressions, regulatory pathways, the prognostic accuracy of *FAM46C* and the correlation of *FAM46C* expression with immune cell infiltration and immune regulation. Our study revealed that *FAM46C* might be a potential clinical prognostic marker, which is closely related to immune regulation, thus providing relevant ideas for developing future tumour immunotherapy.

## Methods

### Clinical Specimens

A total of 20 histological specimens from patients with pathologically confirmed bladder cancer were collected. The specimens comprised 20 pairs of bladder cancer and precancerous tissues. All patients had a confirmed pathological diagnosis before tissue collection and were not treated with chemotherapy or radiotherapy. RNA-sequencing was performed for all specimens. All patients signed an informed consent form. The Medical Ethics Committee of the Affiliated Tumor Hospital of Guangxi Medical University approved the cancer specimen collection protocol. All experiments and methods were performed following relevant guidelines and regulations.

### Data Collection

The RNA expression and clinical data of TCGA and GTEx cohorts were obtained from the UCSC Xena database (https://xenabrowser.net/datapages/). DNA copy numbers were downloaded from the cBioPortal database (https://www.cbioportal.org/).

### Immunohistochemical Staining

All collected cancer and paraneoplastic tissues were fixed in 10% formalin for 48 h, dehydrated in ethanol gradient for 2 h and embedded in paraffin. The tissue specimens were cut into sections of 4–7 µm on a microtome and transferred to slides for drying and maintaining the morphological features of tissue specimens. The slides were dewaxed and hydrated, and the antigen was extracted. The slides were quenched in 3% hydrogen peroxide (H_2_O_2_) solution diluted in distilled water for 10 min at room temperature and incubated with anti-*FAM46C*(Beijing Bioss Biotechnology Co., Ltd. Antibody No.: bs-8198)for 1 h at room temperature. After incubation, the sections were stained with horseradish peroxidase (HRP)-labelled secondary antibody (Shanghai Long Island Biotechnology Co., Ltd.) for 30 min at room temperature. Subsequently, the sections were stained with diaminobenzidine (DAB) for 1 min at room temperature and haematoxylin for 3 min and rinsed with tap water for 10 min. According to the positive rate of tumour cells, the samples were divided into the high (positive rate > 25%) and low expression (positive rate < 25%) groups.

### Differential Expression of *FAM46C*

Oncomine is the largest tumour gene microarray database to date and allows comprehensive data mining. We used the Oncomine database to analyse the differential expression of *FAM46C* mRNA between different tumours and normal tissues. The SangerBox software was used to compare the differences in *FAM46C* expression between cancerous versus paraneoplastic tissues integrated data of TCGA and GTEx databases.

### Genetic Alteration Analysis

The varying genetic characteristics of *FAM46C* were obtained through the cBioPortal website (https://www.cbioportal.org/). We analysed the mutation frequency, copy number variation and mutation type using the “Cancer Type Summary” module of TCGA database and the “Quick Selection” and “TCGA Pan-Cancer Atlas Study” options.

### Survival Analysis

We divided the tissue samples into high and low expression groups based on *FAM46C* mRNA expression values in the dataset, and the prognostic value of *FAM46C* was assessed using Kaplan–Meier survival analysis in both groups. Cox regression analysis was performed using the survive, glmnet, surviveROC and survminer R packages to analyse the correlation of *FAM46C* expression with OS, disease-specific survival (DSS) and progression-free survival (PFS) in different cancer types using TCGA data.

### Immune Infiltration Analysis

We comprehensively analysed the correlation between *FAM46C* expression and immune infiltration using the TIMER2 database and CIBERSORT algorithm. In addition, we analysed the relationship between *FAM46C* and immune cells such as CD4^+^ T cells, CD8^+^ T cells, neutrophils, macrophages and dendritic cells (DCs) at B-cell abundance. The immune, matrix and ESTIMATE scores of *FAM46C* expression were evaluated using the ESTIMATE algorithm.

### Gene Set Enrichment Analysis

We used the clusterProfiler R package for performing gene set enrichment analysis (GSEA) to identify signalling pathways affected by *FAM46C*. Gene sequences obtained based on the fold change of mean gene expression between patients in the high and low *FAM46C* expression groups were entered into a file. Biological processes were evaluated using the Kyoto Encyclopedia of Genes and Genomes (KEGG) and HALLMARK pathway analyses.

### Statistical Analysis

Based on the mRNA expression of *FAM46C*, the samples were divided into a high and low expression group. The Spearman’s correlation was used to assess the correlation between *FAM46C* expression and relevant targets, including tumour mutational burden (TMB) and microsatellite instability (MSI). We used Cox regression analysis to examine hazard ratios (HRs) and log-rank *p* values of survival analyses, and *p* < 0.05 was considered statistically significant.

## Results

### Analysis of *FAM46C* Expression in Pan-Cancer

**FIGURE 1:**
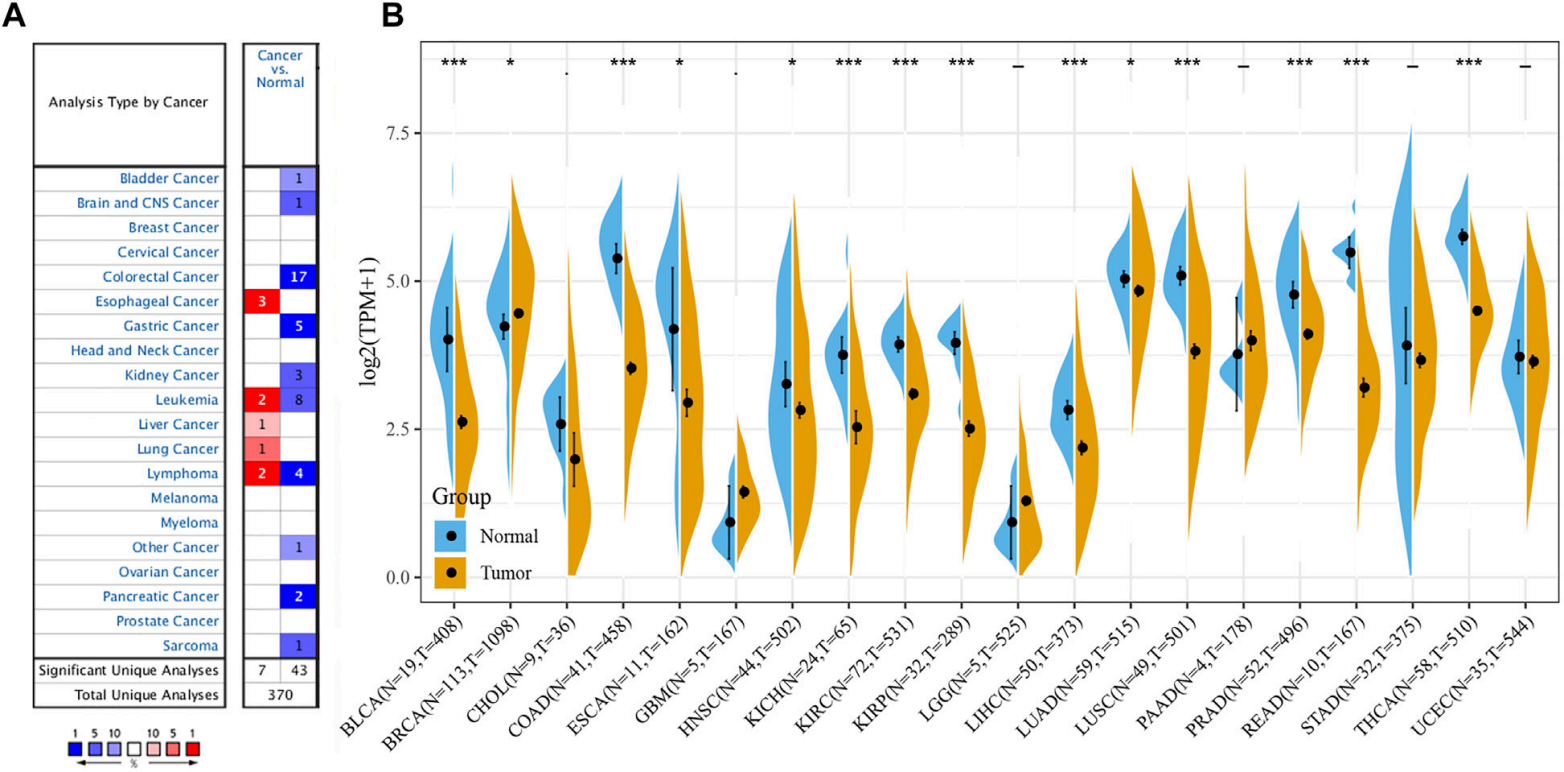
Different levels of *FAM46C* expression in pan-cancer. **(A)** *FAM46C* expression in different cancer and paired normal tissues in the Oncomine dataset. The number in each cell is the number of datasets. **(B)** *FAM46C* expression in tumour tissues from TCGA cohort and normal tissues from TCGA and GTEx cohorts (*p < 0.05, **p < 0.01, ***p < 0.001).

The Oncomine data revealed that the mRNA expression levels of *FAM46C* were significantly decreased in most cancers, such as bladder, brain and central nervous system, colorectal, stomach, kidney and pancreatic cancers; leukaemia and sarcoma (SARC). However, the mRNA expression of *FAM46C* was high in hepatocellular carcinoma and lung cancer (Figure 1A). In addition, considering the small number of normal samples in TCGA, we integrated data from normal tissues in the GTEx database and tumour tissues in TCGA database to analyse the differential expression of *FAM46C* in 20 cancers. Consequently, low *FAM46C* expression was observed in eight tumours: bladder uroepithelial carcinoma (BLCA), colon adenocarcinoma (COAD), oesophageal cancer (ESCA), kidney chromophobe (KICH), prostate adenocarcinoma (PRAD), rectal adenocarcinoma (READ), stomach adenocarcinoma (STAD) and thyroid carcinoma (THCA) (Figure 1B). These results revealed that the mRNA expression of *FAM46C* was low in various tumours, with *FAM46C* playing a protective role in tumour development.

### Relationship Between *FAM46C* Expression and Tumour Stages

**FIGURE 2:**
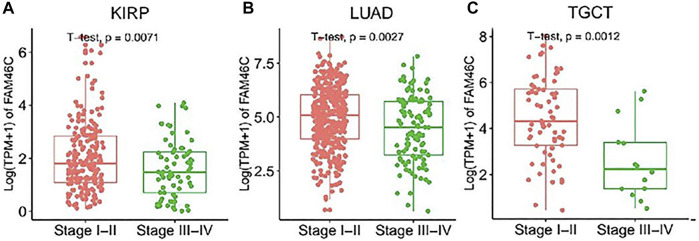
*FAM46C* expression in pan-cancer with different World Health Organizatio stages. **(A–C)** Comparison of early and late expression of *FAM46C* in different tumours.

We further evaluated the expression levels of *FAM46C* in different tumour stages. As shown in Figure 2, the *FAM46C* expression levels are significantly higher in patients with stage I–II disease than in patients with stage III–IV disease in renal papillary cell carcinoma (KIRP), lung adenocarcinoma (LUAD) and testicular germ cell tumour (TGCT) (all *p* < 0.01). However, a decreasing trend in the *FAM46C* expression levels was observed in patients with stage III–IV tumours such as THCA, liver hepatocellular carcinoma (LIHC), lung squamous carcinoma (LUSC), mesothelioma (MESO) and READ; however, the difference was not statistically significant (Supplementary Figure 1).

### Genetic Alterations in *FAM46C*

**FIGURE 3:**
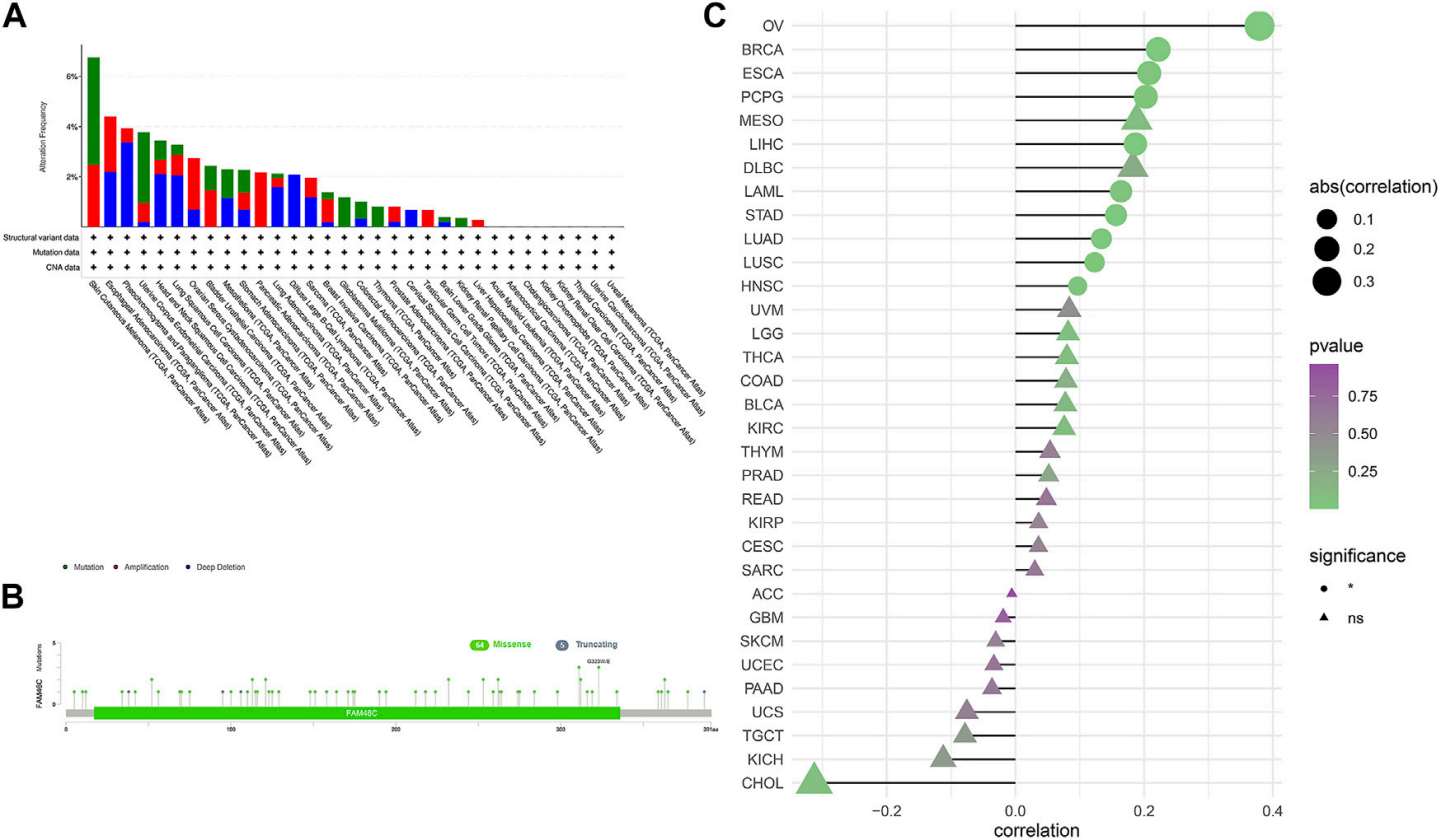
Characterisation of *FAM46C* gene mutations in different TCGA tumour samples as analysed using the cBioPortal tool. **(A)** The type and frequency of *FAM46C* gene mutations in different cancers. **(B)** The mutation sites of *FAM46C*. **(C)** The correlation between copy number changes and *FAM46C* expression in each tumour sample.

We extracted *FAM46C* mutation data from TCGA dataset and used the cBioPortal tool to analyse the extracted data and correlation between *FAM46C* mutations and tumours. As shown in Figure 3A, *FAM46C* has the highest mutation frequency (>6%) in patients with cutaneous melanoma. However, it was less frequently mutated in LIHC, KIRP and low-grade glioma (LGG) of the brain (approximately <1%). Furthermore, the main types of “amplified” copy number alterations (CNAs) were observed in ovarian plasmacytoid cystic adenocarcinoma (OV) and pancreatic adenocarcinoma (PAAD), and *FAM46C* was amplified in all patients with PAAD. The loci and types of *FAM46C* mutation and the number of patients harbouring the mutation are shown in Figure 3B. The main type of genetic alteration in *FAM46C* was missense mutation. As demonstrated in Figure 3C, GSEA reveals that *FAM46C* is positively correlated with DNA copy number in OV, breast invasive carcinoma (BRCA), ESCA, pheochromocytoma and paraganglioma (PCPG), LIHC, acute myeloid leukaemia (LAML), STAD, LUAD, LUSC and head and neck squamous cell carcinoma (HNSC).

### Analysis of the Protein Expression Levels of *FAM46C*

**FIGURE 4:**
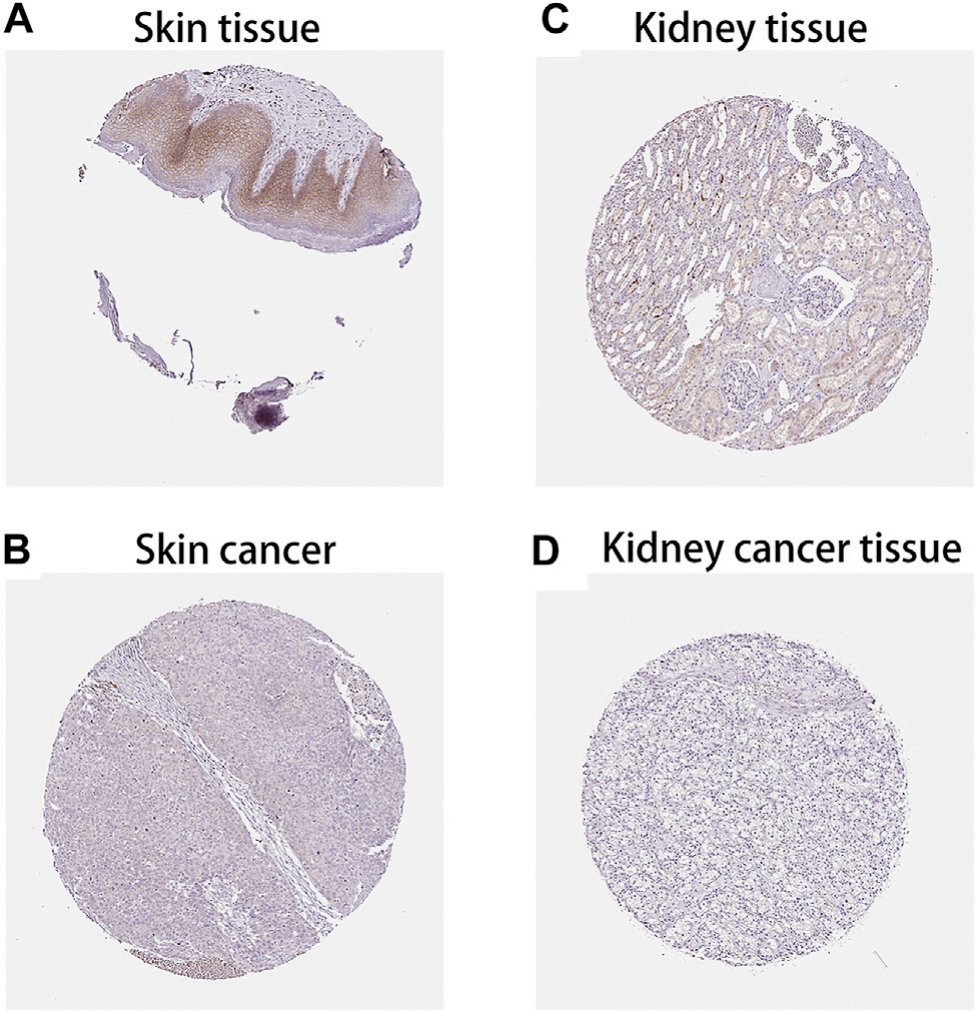
Protein levels of *FAM46C* in tumour and normal tissues from the Human Protein Atlas (HPA) database. **(A,C)** Normal skin and kidney tissues, respectively. **(B,D)** Skin and kidney cancer tissues, respectively.

**FIGURE 5:**
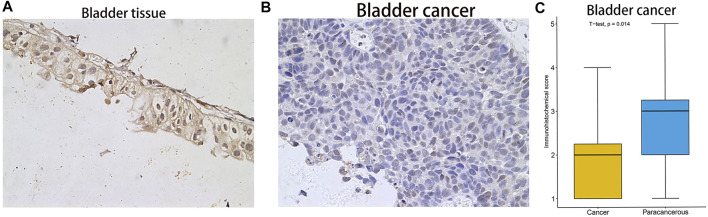
Immunohistochemical staining results and statistical plots of *FAM46C* protein expression in normal bladder and bladder cancer tissues. **(A)** Represent 20× immunohistochemical image of bladder tissue. **(B)** Represent 20× immunohistochemical image of bladder cancer. **(C)** Statistical plots of *FAM46C* protein expression in bladder cancer and normal tissues.

**FIGURE 6:**
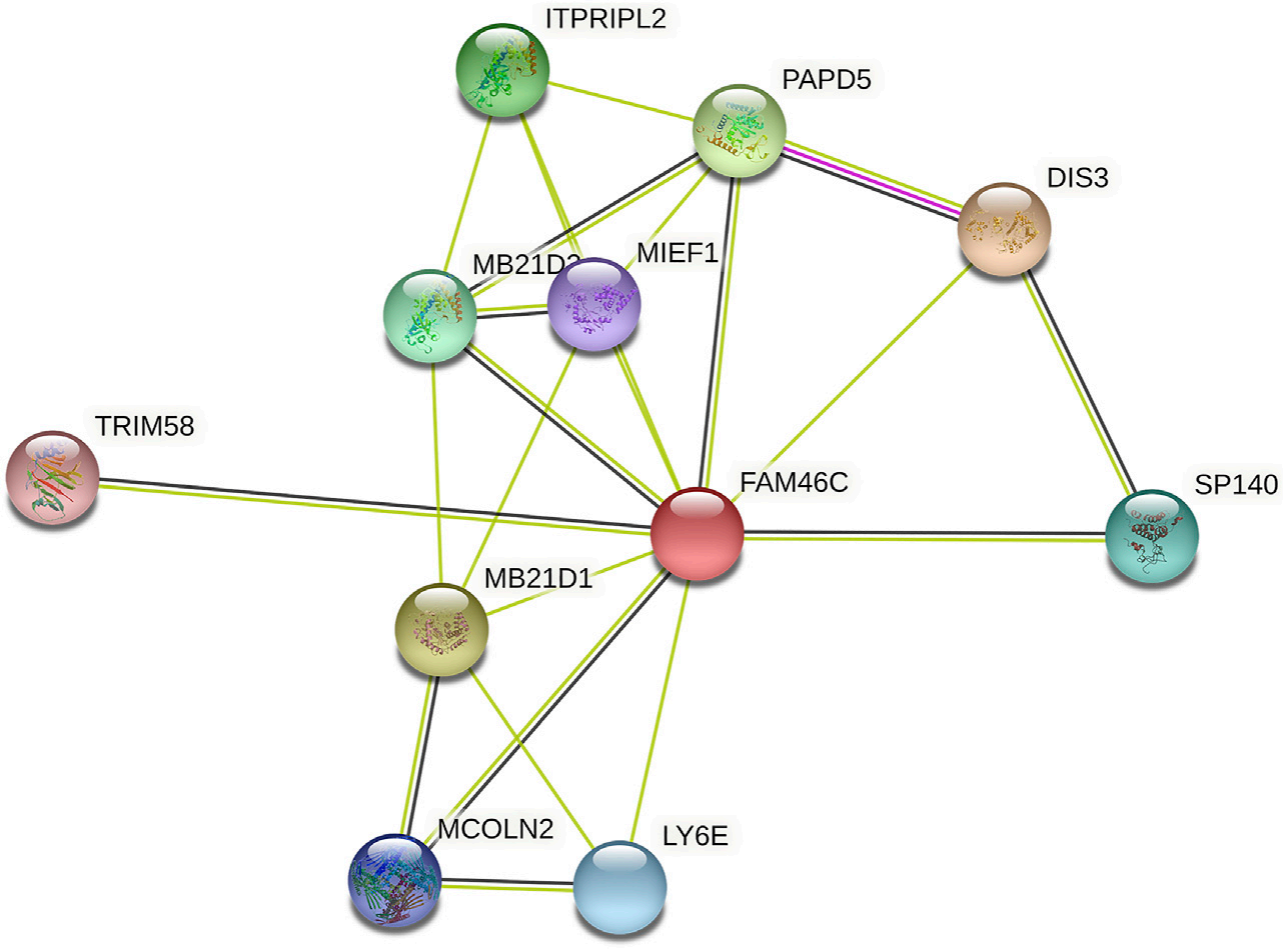
*FAM46C* protein–protein interaction network.

After analysing the mRNA expression, mutations and CNAs of *FAM46C*, we assessed the protein expression level of *FAM46C* in tumours. Using the Human Protein Atlas (HPA) database, we found that the expression level of *FAM46C* was significantly decreased in skin and kidney cancer tissues compared with normal tissues (Figures 4A–D). In addition, we performed immunohistochemical and statistical analyses on paired tissue specimens from patients with bladder cancer, and the results revealed that the expression level of *FAM46C* was significantly decreased in bladder cancer (*p* = 0.014), which was consistent with the analysis of the HPA database (Figures 5A–C). Furthermore, to assess the relationship between *FAM46C* and protein interactions, we constructed a protein–protein interaction (PPI) network and found that *FAM46C* was closely associated with ITPRIPL2, PAPD5, DIS3, SP140, MB21D2, MIEF1, TRIM58, MB21D1, MCOLN2, LY6E and other proteins (Figure 6). Some previous studies have suggested that the SP140 protein may play a role in chromogranin-mediated regulation of gene expression involved in the pathogenesis of LAML (Hoang et al., 2019). However, DIS3 and PAPD5 may act as RNA ectodomain complexes involved in the degradation or transcription of various cellular RNAs (Hu et al., 2020; Liu et al., 2020). Moreover, MIEF1 and LY6E may mediate different signalling pathways to induce apoptosis in the corresponding cancer cells (Yeom et al., 2016; Hou et al., 2019; Zhou et al., 2019). The expression level of TRIM58 protein may be related to the prognosis of patients with LUAD and COAD (Liu et al., 2018; Chen et al., 2021). In addition, the MCOLN2 protein expression level may regulate the development of paediatric acute lymphoblastic leukaemia (Almamun et al., 2015).

### Prognostic Significance of *FAM46C*

**FIGURE 7:**
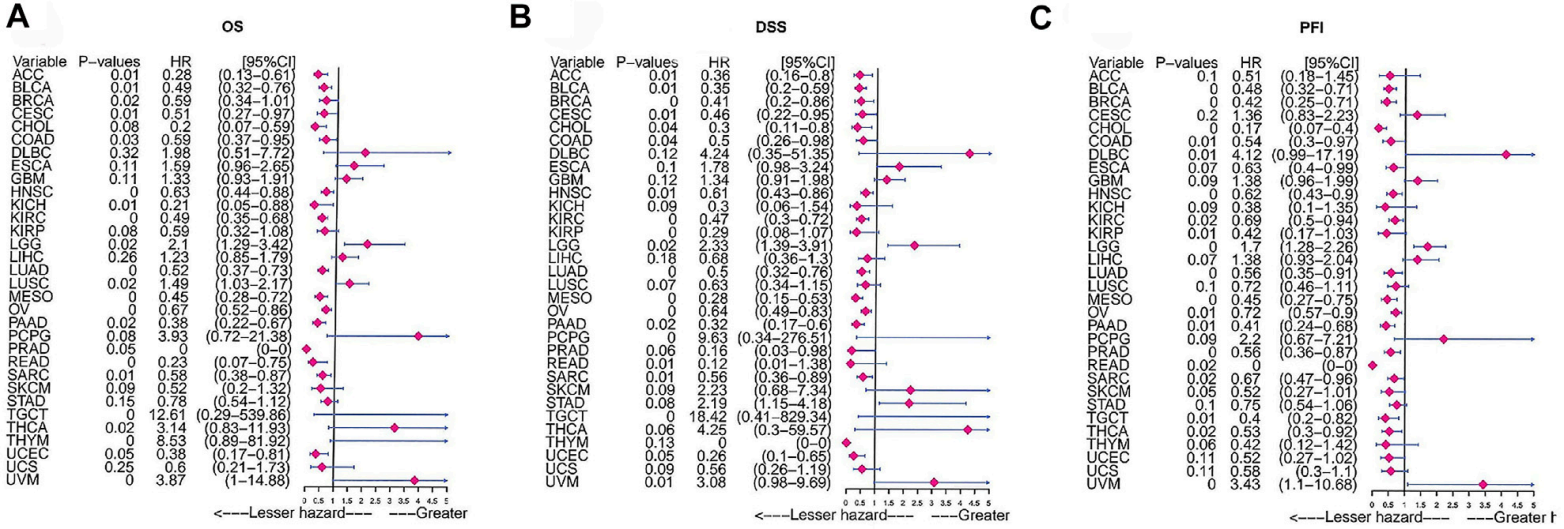
Prognostic analysis of *FAM46C* via univariate Cox regression. The results of **(A)** OS analysis, **(B)** DSS analysis and **(C)** PFI analysis in pan-cancer.

We analysed the clinical value of *FAM46C* using univariate Cox regression and Kaplan–Meier analyses. Univariate Cox regression analysis revealed that high expression of *FAM46C* was associated with a better prognosis. The results of OS analysis revealed that *FAM46C* was associated with improved prognosis in adrenocortical carcinoma (ACC) (*p* = 0.01,HR = 0.28), BLCA (*p* = 0.01, HR = 0.49), BRCA (*p* = 0.02, R = 0.59), cervical squamous cell carcinoma and endocervical adenocarcinoma (CESC) (*p* = 0.01, HR = 0.59), COAD (*p* = 0.03, HR = 0.59), KICH (*p* = 0.01, HR = 0.21), PAAD (*p* = 0.02, HR = 0.38) and SARC (*p* = 0.01, HR = 0.58) (Figure 7A).The DSS analysis revealed that high expression of *FAM46C* was significantly associated with improved prognosis in 11 out of 32 tumours (Figure 7B). According to data on progression-free interval (PFI), high expression of *FAM46C* was significantly associated with prognosis in 12 out of 32 tumours (Figure 7C).

**FIGURE 8:**
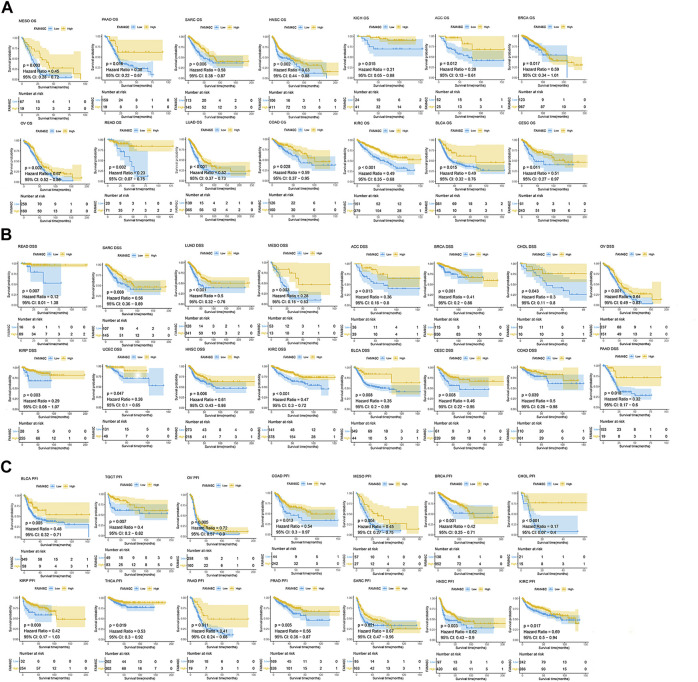
Prognostic analysis of *FAM46C* using Kaplan–Meier curves for **(A)** OS, **(B)** DSS and **(C)** PFI in different tumours.

We analysed the relationship between *FAM46C* expression and OS, DSS and PFI using Kaplan–Meier analysis. The results revealed that high *FAM46C* expression was associated with OS and was a protective factor in ACC (*p* = 0.012, HR = 0.28), BRCA (*p* = 0.017, HR = 0.59), BLCA (*p* = 0.015, HR = 0.49), CESC (*p* = 0.011, HR = 0.51), COAD (*p* = 0.028, HR = 0.59), KICH (*p* = 0.015, HR = 0.21), HNSC (*p* = 0.002, HR = 0.63), renal clear cell carcinoma (KIRC) (*p* < 0.001, HR = 0.49), LUAD (*p* < 0.001, HR = 0.52), MESO (*p* = 0.003, HR = 0.45), OV (*p* = 0.002, HR = 0.67), READ (*p* = 0.002, HR = 0.23), PAAD (*p* = 0.016, HR = 0.38) and SARC (*p* = 0.006, HR = 0.58) (Figure 8A). The DSS analysis revealed that *FAM46C* was the most important protective factor in ACC (*p* = 0.013, HR = 0.36), BRCA (*p* < 0.001, HR = 0.41), cholangiocarcinoma (CHOL) (*p* = 0.043, HR = 0.3), BLCA (*p* = 0.008, HR = 0.35), CESC (*p* = 0.008, HR = 0.46), COAD (*p* = 0.039, HR = 0.5), HNSC (*p* = 0.006, HR = 0.61), KIRC (*p* < 0.001, HR = 0.47), KIRP (*p* = 0.003, HR = 0.29), LUAD (*p* < 0.001, HR = 0.5), MESO (*p* = 0.003, HR = 0.28), OV (*p* = 0.001, HR = 0.64), READ (*p* = 0.007, HR = 0.12), PAAD (*p* = 0.018, HR = 0.32), SARC (*p* = 0.008, HR = 0.56), uterine corpus endometrial carcinoma (UCEC) (*p* = 0.047, HR = 0.26) (Figure 8B). The PFI analysis revealed that *FAM46C* was a protective factor in BRCA (*p* < 0.001, HR = 0.42), CHOL (*p* < 0.001, HR = 0.17), BLCA (*p* = 0.005, HR = 0.48), COAD (*p* = 0.013, HR = 0.54), HNSC (*p* = 0.003, HR = 0.62), KIRC (*p* = 0.017, HR = 0.69), KIRP (*p* = 0.008, HR = 0.42), MESO (*p* = 0.004, HR = 0.45), OV (*p* = 0.005, HR = 0.75), PAAD (*p* = 0.011, HR = 0.41), PRAD (*p* = 0.005, HR = 0.56), SARC (*p* = 0.021, HR = 0.67), TGCT (*p* = 0.007, HR = 0.4) and THCA (*p* = 0.019, HR = 0.53) (Figure 8C).

### Gene Set Enrichment Analysis of *FAM46C*

**FIGURE 9:**
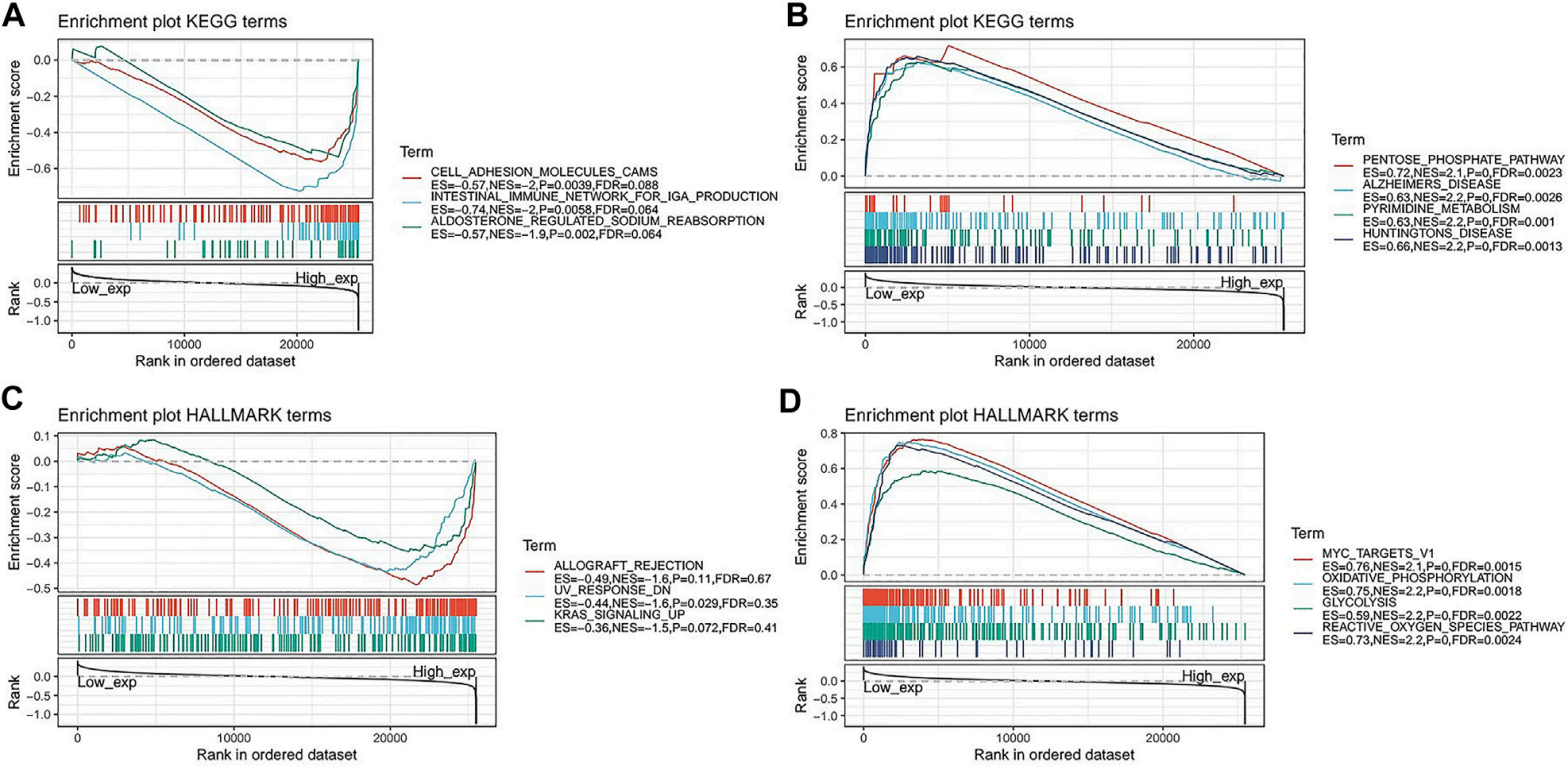
Gene set enrichment analysis of *FAM46C*. Functional terms linked with *FAM46C* enrichment through **(A,B)** KEGG pathway analysis and **(C,D)** HALLMARK pathway analysis.

To further explore the possible signalling pathways involving *FAM46C*, we evaluated 33 tumour types in TCGA database using GSEA, and gene enrichment pathways were analysed using KEGG and HALLMARK. According to KEGG analysis, cell adhesion, intestinal immunity and aldosterone–sodium ion regulation pathways were enriched in high *FAM46C* expression, suggesting that *FAM46C* had regulatory effects on these pathways (Figure 9A). However, biosynthetic pathways such as pentose phosphate and pyrimidine metabolism and pathogenic mechanisms such as those in Alzheimer’s and Huntington’s diseases were enriched in low *FAM46C* expression, suggesting that *FAM46C* might play an inhibitory role in these pathways (Figure 9B). According to the HALLMARK pathway analysis, ultraviolet (UV) receptor signalling pathways were enriched in high *FAM46C* expression, suggesting that *FAM46C* expression was positively correlated with these pathways (Figure 9C). However, oxidative phosphorylation, reactive oxygen metabolism and pathways such as *MYC* genes and glycolysis were enriched in low *FAM46C* expression, suggesting that *FAM46C* expression was negatively correlated with these pathways owing to its inhibitory effects (Figure 9D).

### Immune Cell Infiltration Analyses

**FIGURE 10:**
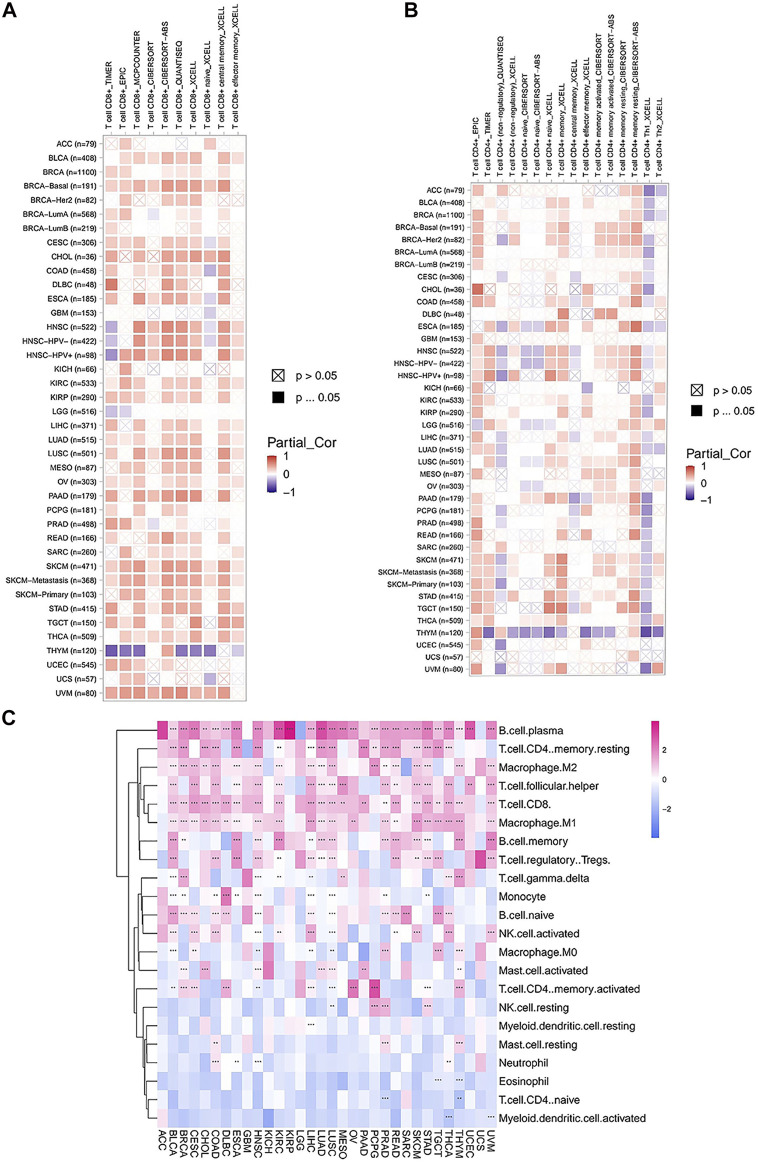
Correlation analysis between *FAM46C* expression and immune infiltration of cells. Correlation of *FAM46C* expression with **(A)** CD8^+^ T-cell infiltration, **(B)** CD4^+^ T-cell infiltration and **(C)** different types of immune infiltrating cells in pan-cancer.

To assess the relationship between *FAM46C* expression and immune cell infiltration, we used two different sources of immune cell infiltration data for correlation analysis. The TIMER2 database results revealed that in most tumours, *FAM46C* was positively correlated with CD4^+^ and CD8^+^ T-cell infiltration levels and negatively correlated with CD4^+^ Th1-cell infiltration levels (*p* < 0.05) (Figures 10A,B). We performed correlation analysis on published data using the CIBERSORT algorithm and evaluated 26 immune cells. The results revealed that *FAM46C* was positively correlated with the infiltration levels of plasma B cells, regulatory memory CD4^+^ T cells and CD8+T cells but was negatively correlated with the infiltration levels of bone marrow DCs and naive CD4^+^ T cells (*p* < 0.05) (Figure 10C). These results suggested that *FAM46C* promoted T-cell infiltration, thus explaining the protective role of *FAM46C* in most tumours.

### Correlation of *FAM46C* With Immunomodulation-Related Genes and Chemokines

The association between *FAM46C* and immune-regulated genes in pan-cancer was observed using the Spearman’s correlation based on the stromal, immune and ESTIMATE scores (*p* < 0.05 and |R| > 0.3). *FAM46C* expression was specifically correlated with stromal fraction in HNSC (R = 0.4, *p* < 0.05), LGG (R = 0.395, *p* < 0.05) and LUSC (R = 0.592, *p* < 0.05) (Supplementary Figure 2). In addition, a specific correlation was observed between immune scores and *FAM46C* in BLCA (R = 0.443, *p* < 0.05), COAD (R = 0.539, *p* < 0.05), diffuse large B-cell lymphoma (DLBC) (R = 0.486, *p* < 0.05), HNSC (R = 0.577, *p* < 0.05), LGG (R = 0.415, *p* < 0.05), LUSC (R = 0.671, *p* < 0.05), skin cutaneous melanoma (SKCM) (R = 0.595, *p* < 0.05), TGCT (R = 0.559, *p* < 0.05) and uterine carcinosarcoma (UCS) (R = 0.359, *p* < 0.05) in which there was (Supplementary Figure 3). In addition, a higher correlation was observed between *FAM46C* and ESTIMATE scores in BLCA (R = 0.44, *p* < 0.05), COAD (R = 0.463, *p* < 0.05), DLBC (R = 0.386, *p* < 0.05), HNSC (R = 0.548, *p* < 0.05), LGG (R = 0.42, *p* < 0.05), LUSC (R = 0.666, *p* < 0.05), MESO (R = 0.33, *p* < 0.05) and SKCM (R = 0.589, *p* < 0.05), which was used as a combined ratio to assess the immune and stromal components in tumour tissues (Supplementary Figure 4).

**FIGURE 11:**
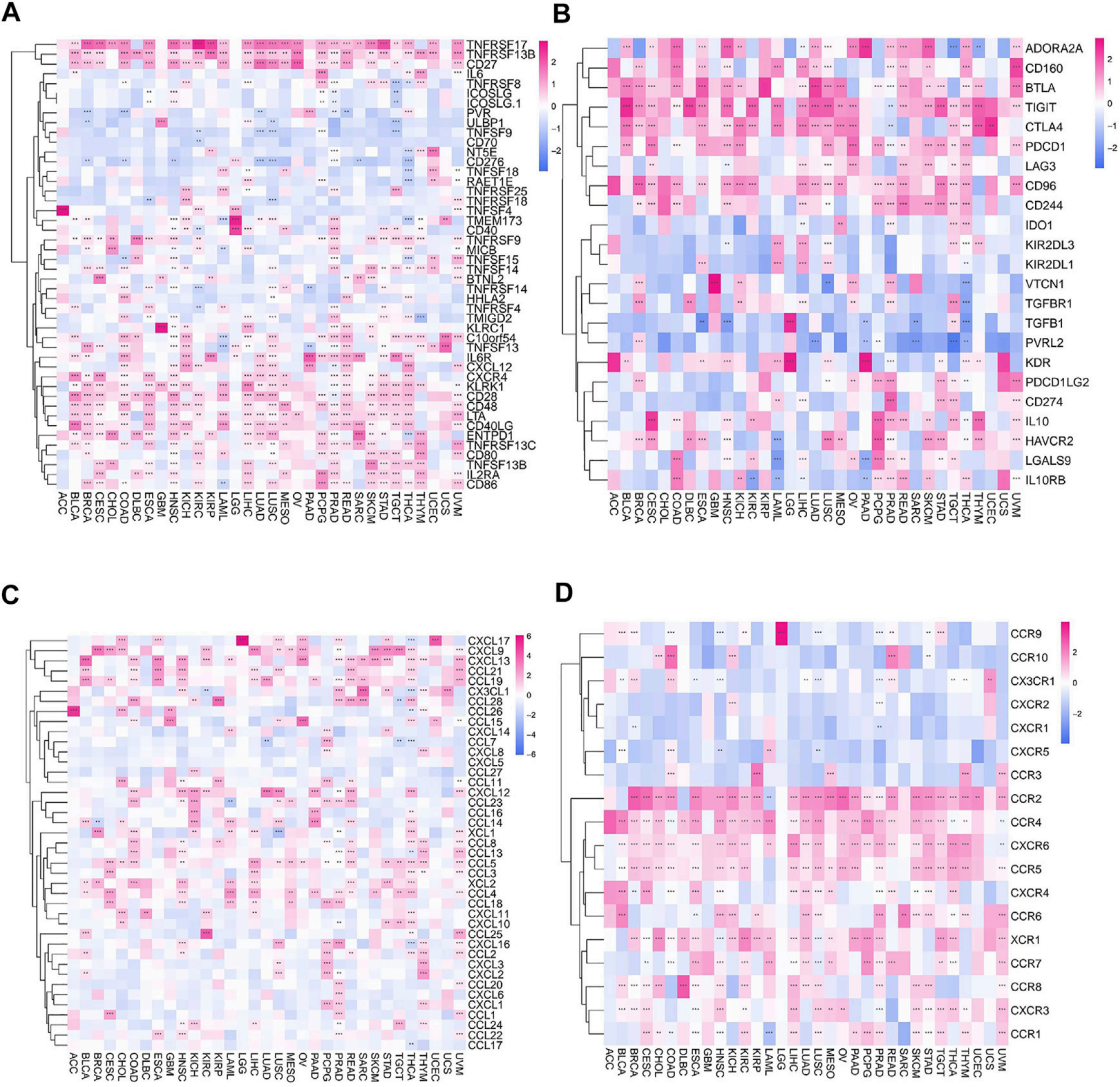
Relationship between *FAM46C* expression and immune-related factors. Relationship between *FAM46C* expression and **(A)** immune activating genes, **(B)** immunosuppressive genes, **(C)** immune chemokines and **(D)** immune chemokine receptors (*p < 0.05, **p < 0.01, ***p < 0.001).

Furthermore, the correlation between *FAM46C* and immune activation genes in pan-cancer was analysed. *FAM46C* expression was found to be positively correlated with TNFRSF17 and TNFRSF13B, except in DLBC, thymoma (THYM) and UCS. In most human tumours, *FAM46C* was negatively correlated with CD70. In addition, we also found that *FAM46C* expression exhibited a more significant negative correlation with CD276 and TNFSF9 in LUAD and LUSC (both *p* < 0.01) (Figure 11A). Immunosuppression also plays an important role in the immune regulation of tumours. The results revealed that *FAM46C* expression was positively correlated with TIGIT and negatively correlated with PVRL2 in most tumours, especially in LUAD, SARC and TGCT (all *p* < 0.01) (Figure 11B). We also investigated the specific association between *FAM46C* expression and chemokines and their receptors in pan-cancer and found that *FAM46C* expression was positively correlated with immune chemokines (CXCL9 and CXCL13) and immune chemokine receptors (CCR2 and CCR4) in most tumours (all *p* < 0.01) (Figures 11C,D).

### *FAM46C* Expression Is Related to Immune Checkpoint Genes in Human Cancers

**FIGURE 12:**
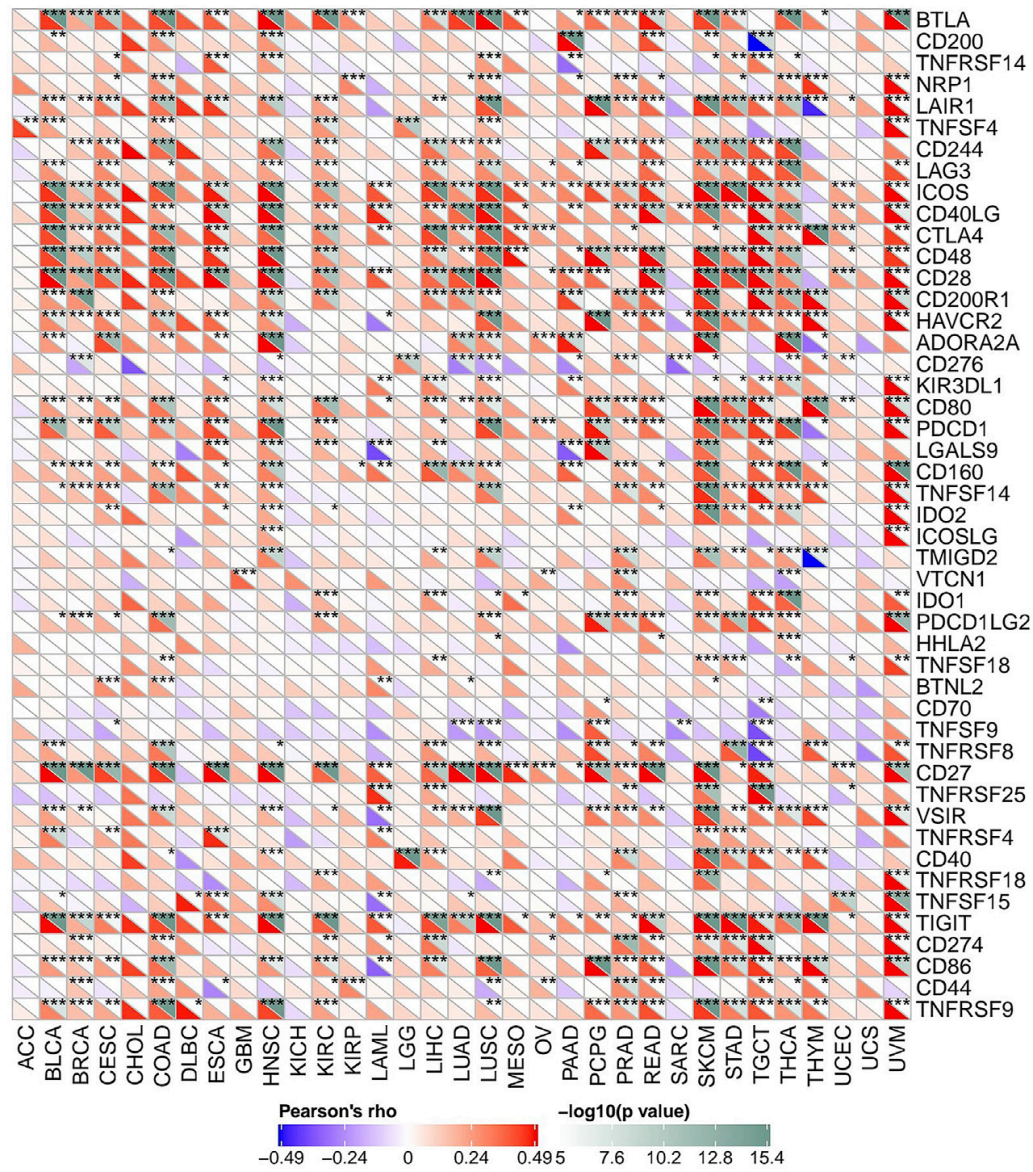
The association between *FAM46C* expression and immune checkpoint genes in pan-cancer (*p < 0.05, **p < 0.01, ***p < 0.001).

As mentioned above, we explored the role of *FAM46C* in immunomodulation. Currently, immunotherapy is one of the important tumour treatment approaches. Immunotherapy controls tumour progression by restarting and maintaining the tumour–immunity cycle and restoring the normal anti-tumour immune response of the body. Previous studies have demonstrated the significant effect of immune checkpoint (ICP) genes on immunotherapy (Zerfas et al., 2020). To further explore the potential of *FAM46C* in immunotherapy, we analysed the relationship between *FAM46C* expression and ICP genes. As demonstrated in Figure 12, *FAM46C* expression is positively correlated with 47 ICP genes in BLCA, BRCA, CESC, COAD, ESCA, HNSC, KIRC, LAML, LIHC, LUAD, LUSC, PAAD, PCPG, PRAD, READ, SKCM, STAD, TGCT, THCA, THYM and uveal melanoma (UVM). In particular, more than 30 of the 47 ICP genes were associated with *FAM46C* expression in COAD, SKCM, HNSC and UVM. In addition, *FAM46C* expression exhibited a positive correlation with several tumour types, suggesting that *FAM46C* promotes ICP gene activation by participating in several different signalling pathways and is a potential immunotherapeutic target (Figure 12).

### Relationship Between *FAM46C* Expression and TMB and MSI

**FIGURE 13:**
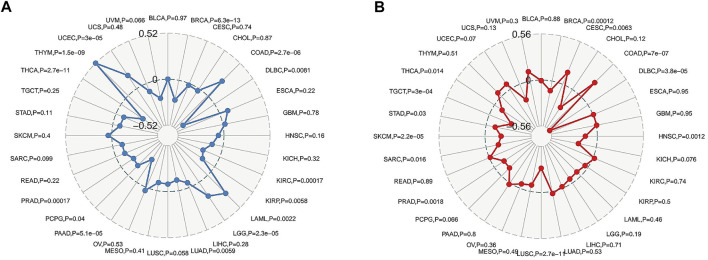
Relationship between *FAM46C* gene expression and **(A)** TMB and **(B)** MSI in pan-cancer.

TMB is an indicator of the extent of mutations in tumour cell species (Nikanjam et al., 2018). MSI, caused by defects in mismatch repair (MMR) genes, is closely associated with tumorigenesis and is an important molecular marker for the prognosis of solid tumours and the development of adjuvant immunotherapy regimens (Srinivas et al., 2001). The relationship between FAM46C expression and TMB and MSI can be evaluated for determining the potential clinical feasibility of immunisation with FAM46C. We used the Spearman’s correlation to assess the correlation between FAM46C expression and TMB and MSI. As demonstrated in Figures 13A,B, FAM46C is positively correlated with high TMB in LAML (*p* = 0.0022) and high MSI in CESC (*p* = 0.0063).

## Discussion

The *FAM46C* gene is widely expressed in animal genomes, and its regulation encodes a non-canonical polymerase. Previously, mutations in the *FAM46C* gene were reported to be associated with the development and invasion of myeloma, with frequent mutations in *FAM46C* affecting the outcome and clinical prognosis of patients with myeloma. However, in recent years, abnormal expression levels of *FAM46C* have been found in several cancers. *FAM46C* overexpression inhibits invasion and metastasis and induces cell cycle arrest in hepatocellular carcinoma, whereas *FAM46C* deletion is associated with tumour progression and poor prognosis. In a study, *FAM46C* expression was significantly reduced in colorectal cancer tissues and increased after norethindrone (NCTD) treatment (Wan et al., 2017). These studies suggest that *FAM46C* is functionally associated with various human tumours, which guided us to explore the correlation of *FAM46C* expression levels with immunity, tumour staging and prognosis in various cancers to determine the potential of *FAM46C* in predicting clinical prognosis and immunotherapy. Therefore, we conducted a comprehensive study on FAM46 expression levels in different cancers using the Oncomine, TCGA and TIMER2 data.

First, we assessed the mRNA expression levels of *FAM46C* in tumours in the Oncomine and TCGA datasets. The results revealed that the expression level of *FAM46C* was significantly decreased in most cancers. Furthermore, we evaluated the mRNA expression levels of *FAM46C* in different stages of the same tumour based on TCGA clinical data. The results suggested a decreasing trend of *FAM46C* expression levels in patients with stage III–IV disease in most tumours. In addition, we used univariate Cox regression and Kaplan–Meier analyses to investigate the clinical prognostic value of *FAM46C* expression levels in tumours. It has been reported that reduced *FAM46C* expression levels are associated with shorter OS in patients with gastric cancer (Tanaka et al., 2017). We considered that the study endpoint of OS includes death from non-cancerous causes, which has limitations in reflecting tumour biological behaviour and treatment. To more effectively reflect the clinical factors affecting patients, we analysed the data related to PFI and DSS, and the combined results revealed that the prognosis of most tumours correlated with *FAM46C* expression levels. In particular, in BRCA, BLCA, KIRC, LUAD, MESO, OV, PAAD, SARC and other tumours, the prognosis of patients in the high *FAM46C* expression group was significantly better than that in the low expression group. Therefore, it can be tentatively concluded that *FAM46C* plays a protective role in most tumours.

In previous studies, mutations and changes in the protein expression levels of *FAM46C* have been mostly reported in multiple myeloma. However, the mutations and protein expression levels of *FAM46C* were associated with various tumours in our study. Recent studies have demonstrated that gene mutations alter gene expression levels in tumours and induce changes in the tumour microenvironment (TME). Genes may also bind to new loci to adapt to the new microenvironment via genetic recombination (Li et al., 2019). We found that mutations in the *FAM46C* gene affected its expression levels, which was further associated with altered biological behaviour in various tumours, indicating that *FAM46C* has the potential to predict prognosis and treatment outcomes in patients with tumours. In addition to significantly decreased *FAM46C* mRNA expression levels in most tumours, the expression levels of *FAM46C* and related proteins exhibited a decreasing trend in most tumours. It has been demonstrated that deletion of some genes in tumour cells results in reduced mRNA and protein abundance, which leads to increased tumour cell migration (Herrero et al., 2020), suggesting that the expression levels of related genes and proteins are related to tumorigenesis and invasion. On this basis, we compared our paired immunohistochemical and statistical results in cancerous and paracancerous tissues in the HPA database, and the paired results showed consistency. Immunohistochemical comparison validated the potential of *FAM46C* as a prognostic marker for tumours. In addition, the PPI network indicated that *FAM46C* was involved in regulating the functions of various proteins that contribute to apoptosis and immune recognition of the corresponding cancer cells by mediating different signalling pathways.

The reciprocal protein relationships and differential expression of mRNA reflect the potential value of *FAM46C* in predicting prognosis and modulating immunity. To assess the clinical application of *FAM46C* and the practicality and feasibility of tumour treatment, we analysed the relationship between *FAM46C* and immune pathways, cellular infiltration, immunomodulatory factors and immune checkpoints. According to the KEGG and HALLMARK pathway analyses, *FAM46C* was enriched in different immune pathways, and the expression level of *FAM46C* was closely related to the regulation of different immune and metabolic pathways. A study elucidated that the intestinal immune system is a signalling pathway that produces IgA and inhibits the proliferation and migration of hepatocellular carcinoma (Yang et al., 2018), which is consistent with our findings that the signalling pathway was enriched in high *FAM46C* expression, suggesting that *FAM46C* contributes to this pathway. Previous studies have reported that over-activation of the pentose phosphate pathway plays an important role in promoting the growth of cancer cells (Jiang et al., 2014; Ge et al., 2020). Therapeutic strategies to modulate and balance the pentose phosphate pathway are valuable for tumour treatment. Furthermore, MYC migration and overexpression may lead to increased aggressiveness of related tumours (Walker et al., 2014). In our study, the pentose phosphate and MYC pathways were enriched in low *FAM46C* expression, which further demonstrated the tumour suppressive role of *FAM46C*. This finding indicates that *FAM46C* can positively regulate immune responses and related metabolic pathways to exert a protective role in tumours.

Our study also highlights that *FAM46C* is closely associated with immune cell infiltration. Our results revealed that *FAM46C* was positively correlated with CD4^+^ T cell-, CD8^+^ T cell- and plasma B-cell infiltration levels and negatively correlated with naive CD4^+^ T-cell infiltration levels. A study demonstrated that *FAM46C* expression was decreased and the threshold of T-cell activation was increased in mice infected with Andes virus (ANDV), and *FAM46C* was one of the immune regulatory genes involved in the functioning of the immune system (Campbell et al., 2015). Seweryn Mroczek et al. found that B lymphocyte proliferation was significantly faster after strong induction of *FAM46C* protein in *FAM46C*-FLAG knockout mice, and *FAM46C* functioned as a tumour suppressor in multiple myeloma (Mroczek et al., 2017). Our findings are consistent with their findings, which suggest that *FAM46C* is associated with the production of B and T cells.

For immune stimulators and suppressors, our findings indicate that *FAM46C* is associated with multiple immune drivers in most tumours. Previous studies have reported that CD70 is overexpressed in various tumours. Elevated levels of CD70 expression enhance the suppressive effect of TME on the immune system (Jacobs et al., 2015). Anti-CD70 therapy has emerged as a new tumour immunotherapy approach. A study validated the efficacy of anti-CD70 in glioblastoma, wherein anti-CD70 treatment contributed to the regression of glioblastoma (Jin et al., 2018). However, other tumours that we analysed in this study, such as LUAD and THCA, have not yet been experimentally validated, which will be a target for our future study. However, the correlation between *FAM46C* expression and immunomodulators suggests that *FAM46C* has the potential to predict tumour progression and guide immunotherapy.

In addition, our study found that *FAM46C* expression was closely associated with ICP genes in most tumours, especially in COAD, SKCM, HNSC and UVM. The abnormal expression level of ICP genes in tumour cells is an important reason for the incomplete activation of T cells, which may explain the suppressive effect of TME on the immune response (Zerfas et al., 2020). In previous clinical trials, cancer vaccines have been effective in the treatment of prostate cancer (Jafari et al., 2020), although they could not overcome the suppressive effect of TME on the immune system owing to the weak sensitivity of monoclonal antibodies to immune loci. However, a considerable number of studies have confirmed that ICP genes play a key role in promoting the initiation and progression of tumours and inhibition of dominant immune cells in TME. However, most patients are resistant to a single ICP inhibitor. Therefore, identification of more promoters related to ICP therapy is the future direction of tumour treatment. Based on the findings of previous studies and our study, *FAM46C* can be considered a factor associated with ICP immunotherapy, which may be an ideal treatment strategy in the future.

According to TMB and MSI analyses, high expression of *FAM46C* was associated with LAML and CESC. Higher levels of TMB were found in patients with LAML, with the effectiveness of immunotherapy being higher in this group of patients (Galanina et al., 2018). This finding indicates that TMB is one of the methods to evaluate immunotherapy and also highlights the need to monitor gene mutations in patients with haematological tumours. It has also been reported in the literature that endometrial and cervical cancers in the presence of MSI are better treated with PD-1 immunotherapy (Bonneville et al., 2017), and treatment with immune checkpoint inhibitor is a promising therapeutic approach for future individualised testing. Therefore, the regulation of *FAM46C* is effective in tumours with high MSI.

However, despite the comprehensive and systematic analysis of *FAM46C* using different databases and statistical methods for cross-validation, this study has some limitations. First, bias and discrepancies between different databases may lead to systematic bias. Second, *in vivo*/*in vitro* experiments are required to validate the potential function of *FAM46C* and improve the credibility of our conclusions. Third, although our findings support that *FAM46C* expression is associated with immune cell infiltration and clinical prognosis in pan-cancer, we lack direct evidence for demonstrating the prognostic effect of *FAM46C* on immune infiltration. Therefore, the exact underlying immunomodulatory mechanisms of *FAM46C* remain to be further investigated. Fourth, the effect of *FAM46C* expression levels on prognosis and targeted therapies should be validated in clinical trials. In conclusion, the current role of *FAM46C* in immune infiltration in pan-cancer requires further validation by prospective studies to further guide the clinical treatment of tumours.

## Conclusion

*FAM46C* can be used as a potential prognostic marker for pan-cancer, is closely related to immune regulation and may become an immune checkpoint to guide future clinical immunotherapy.

## Data Availability

The original contributions presented in the study are included in the article/Supplementary Material, further inquiries can be directed to the corresponding author.

## Supplementary Material

Supplementary Figure 1
*FAM46C* expression in pan-cancer with different World Health Organization stages. The differential expression of *FAM46C* is not statistically significant (*p* > 0.05) in the WHO staging of different cancer types in TCGA database.

Supplementary Figure 2 Analysis of the association between *FAM46C* expression and stromal scores using the Spearman’s rho test. The data with *p* < 0.05 are plotted.

Supplementary Figure 3 Analysis of the association between *FAM46C* expression and immune scores using the Spearman’s rho test. The data with *p* < 0.05 are plotted.

Supplementary Figure 4 Analysis of the association between *FAM46C* expression and the ESTIMATE scores using the Spearman’s rho test. The data with *p* < 0.05 are plotted.
