# Supplementary figures and images for: FAM46C as a Potential Marker for Pan-Cancer Prognosis and Predicting Immunotherapeutic Efficacy
Source: Front Genet. 2022 Feb 9;13:810252. doi: 10.3389/fgene.2022.810252 (PMC8864238; doi:10.3389/fgene.2022.810252)

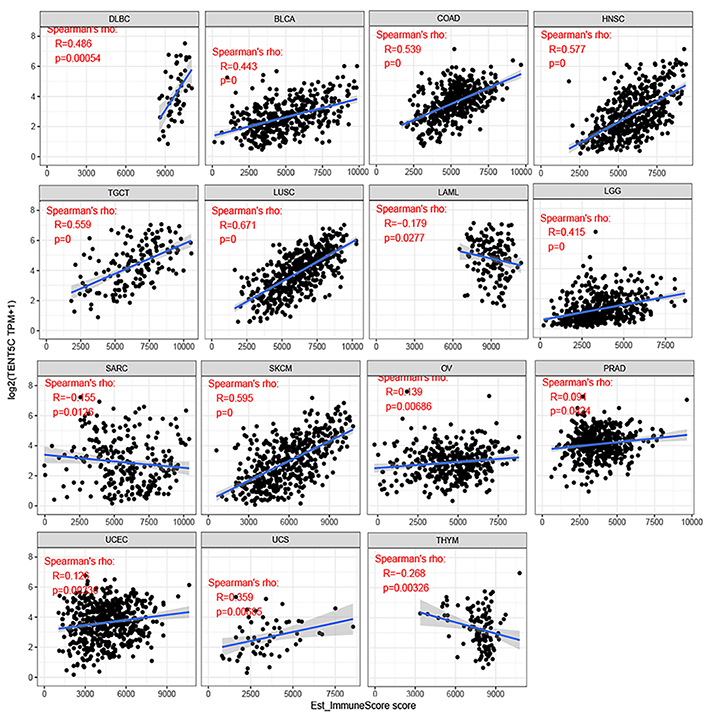

Supplement: Supplementary file 1 [file Image3.TIF]

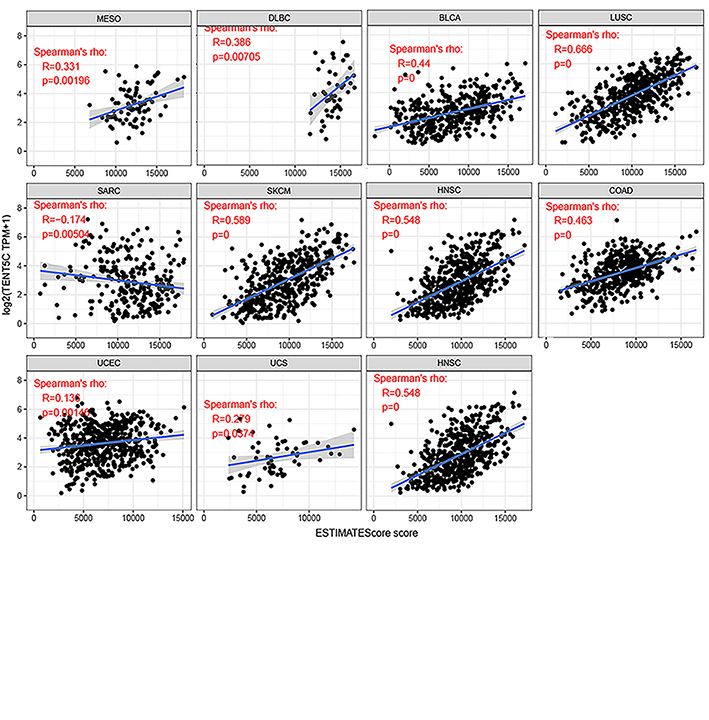

Supplement: Supplementary file 2 [file Image4.TIF]

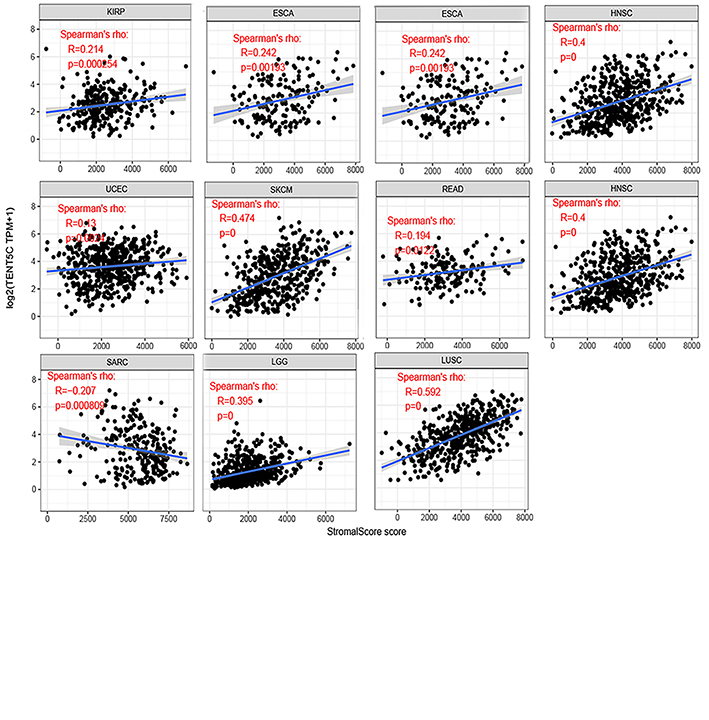

Supplement: Supplementary file 3 [file Image2.TIF]

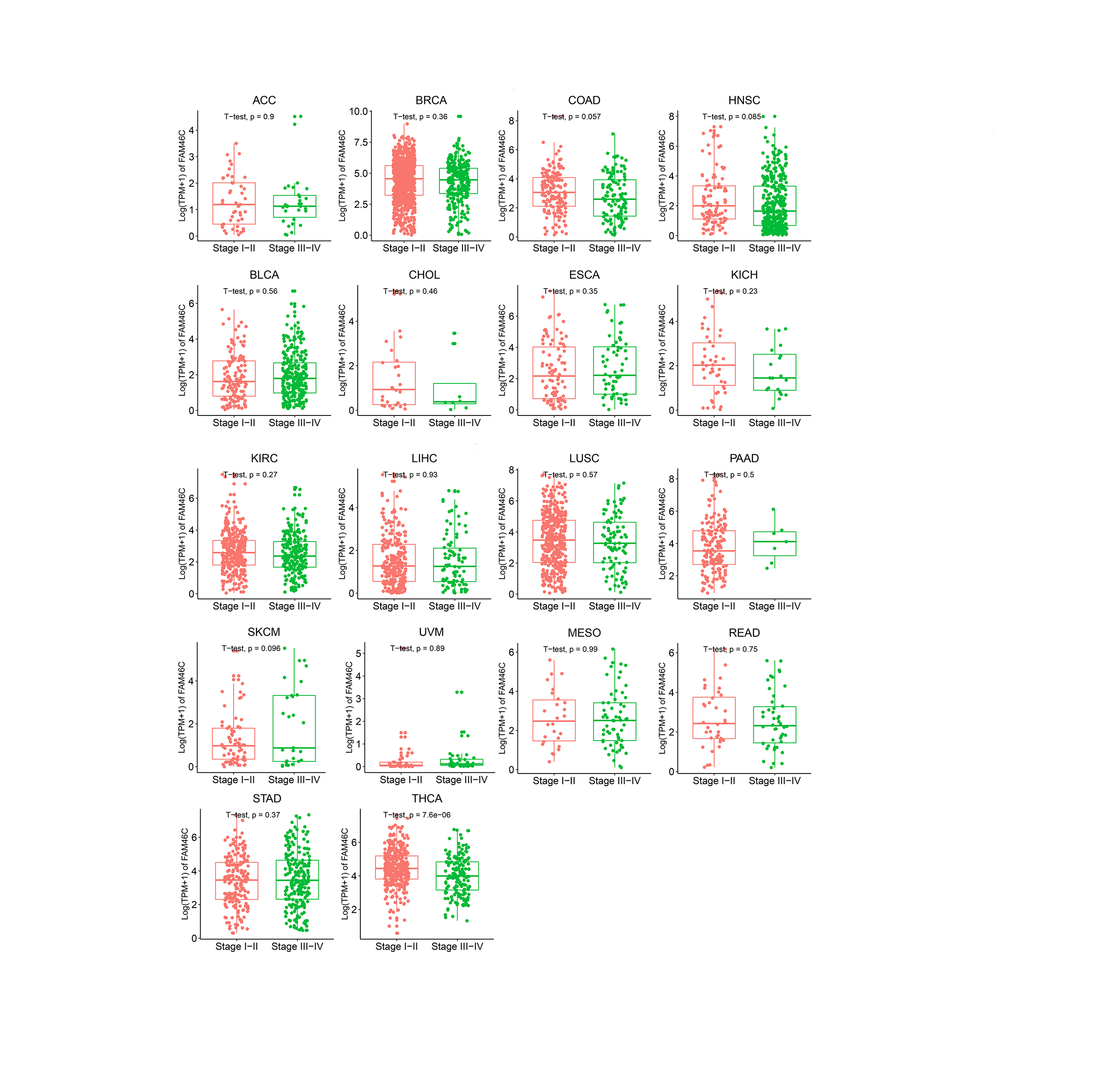

Supplement: Supplementary file 4 [file Image1.TIF]
